# Host–pathogen interaction unveiled by immune, oxidative stress, and cytokine expression analysis to experimental *Saprolegnia parasitica* infection in Nile tilapia

**DOI:** 10.1038/s41598-023-36892-w

**Published:** 2023-06-19

**Authors:** Samar A. El Gamal, Rawia Saad Adawy, Viola Hassan Zaki, Eman Zahran

**Affiliations:** 1 Department of Aquatic Animal Medicine, Faculty of Veterinary Medicine, Mansoura University, Mansoura, 35516 Egypt; 2 Department of Fish Diseases, Animal Health Research Institute (AHRI), Mansoura branch, Agriculture Research Center (ARC), Giza , Egypt

**Keywords:** Diagnostic markers, Ichthyology

## Abstract

The present study evaluated the pathogenicity, immunological, and oxidant/antioxidant responses against *Saprolegnia parasitica* (*S. parasitica*) infection in Nile tilapia (*Oreochromis niloticus*). Three groups of Nile tilapia were assigned as the control group (no zoospores exposure). The other two groups were challenged by Saprolegnia zoospores; one was used for sampling, and the other for mortality monitoring. The study lasted 3 weeks and was sampled at three point times at 1, 2, and 3 weeks. Results showed that *S. parasitica* zoospores were pathogenic to Nile tilapia, causing a cumulative mortality rate of 86.6%. Immunoglobulin M and C- reactive protein (IgM and CRP) levels showed a similar trend being significantly (*P* < 0.05, *P* < 0.001) higher in the infected group at weeks 1, 2, and 3, respectively, compared to the control group. Oxidant and antioxidant parameters in gills revealed that Malondialdehyde (MDA) level was significantly higher in the infected group compared to the control group. While catalase, glutathione peroxidase, and superoxide dismutase (CAT, GSH, and SOD) levels were significantly decreased in the infected group compared to the control group. Compared to the control, the tumor necrosis factor-α (*TNF-α*) gene was firmly upregulated in gill tissue at all-time points, particularly at day 14 post-infection. Meanwhile, Interleukin 1-β *(IL-1 β*) gene was significantly upregulated only at days 7 and 14 post-infection compared to control. Histopathological examination revealed destructive and degenerative changes in both skin and gills of experimentally infected Nile tilapia. Our findings suggest that Nile tilapia-*S. parasitica* infection model was successful in better understanding of pathogenicity and host (fish)-pathogen (oomycete) interactions, where the induced oxidative stress and upregulation of particular immune biomarkers in response to *S. parasitica* infection may play a crucial role in fish defense against oomycetes in fish.

## Introduction

Saprolegniaisis due to *Saprolegnia* oomycetes infection is one of the most important mycotic diseases affecting tilapia aquaculture leading to massive mortalities with severe economic losses^1–3^. Tilapias are Egypt's most important freshwater cultured species due to their faster growth rate, resistance to adverse conditions, and suitability for biological studies^4,5^. However, to meet the population demands, intensification of aquaculture has been adopted. Besides farm management, stocking densities are critical for fish farm productivity; thus, sub-optimal levels adversely affect fish, rendering them susceptible to diseases^6,7^.

*Saprolegnia* spp. are ubiquitous inhabitant of the aquatic environment and are considered secondary invaders associated with stressors, which lower fish immunity and resistance to pathogens^8–10^. *Saprolegnia* infections are more common in cultured fish when certain environmental conditions are met, such as a sudden drop in temperature below 8 °C for an extended period and an increased water pH (> 9)^2,11^. *Saprolegnia parasitica* and *diclina* are the most pathogenic species characterized by mycelial patches attached mainly to the skin and fins. Additionally, the disease spreads through the motile secondary zoospores, which is the infective stage^12,13^. Infection with *Saprolegnia* is characterized histopathologically by degeneration of epidermal cells, localized dermal edema, and eventual sloughing of the epidermis^14,15^.

The immune system involves biological mechanisms that protect living organisms from invading pathogens. Fish immune responses are mediated by diverse cells and secreted soluble mediators, which act together for complete protection^16–18^. Lysozymes, Immunoglobulin M (IgM) are usually the innate immune parameters in fish associated with disease resistance^19^.

Oxidative stress biomarkers are becoming increasingly important in the field of ecotoxicology. The occurrences of pathogens in various animals, including humans, generate reactive oxygen species (ROS), which initiate oxidative damage. The antioxidants protect fish against the released ROS, which is linked not only with the initiation of infection but also with the induction of severe inflammation through the induction of pro-inflammatory cytokines, particularly *TNF-α* and *IL-1*, *IL-4*, *IL-6*, and interferon-gamma (*INF-γ*)^20^. Such cytokine genes are considered immune response biomarkers^21^. They are also involved in different levels of the fish immune system, such as regulation and strengthening of the growth and function of immune cells^3,22^.

Many studies have been concerned with treatment trials to control saprolegniasis; others have focused on the host-immune response during *Saprolegnia* infections; however, the latter mainly targeted salmonids, trouts, and fish cell lines^23^. The host-immune response in Nile tilapia infected with *Saprolegnia* spp. contributing to its pathogenicity has not been much focused as it should be. Consecutive changes in the innate immune and oxidative stress responses are linked to the pathogenicity and are evident in the host–pathogen interactions. Therefore, this study was designed to investigate comprehensive arrays of selected biomarkers involved in the host immune response of Nile tilapia experimentally infected with *S. parasitica*.

## Materials and methods

### Induction of *S. parasitica* zoospore

*Saprolegnia. parasitica* was one of the isolates sexually and molecularly characterized in our recent study^24^. A stock culture of *S. parasitica* was kept on glucose yeast extract agar (GYE) at 19 °C. Zoospores of *S. parasitica* were induced as described elsewhere^25,26^. After zoospores induction, about 120 mL of zoospore suspension was collected from about six Petri dishes containing sterile MSM/group and placed into six plastic aquarium bags for challenge into each aquarium/group.

### Experimental fish

A total of ninety healthy Nile tilapia 50 g on average was transferred alive from a private fish farm in Kafr El-sheikh province to Animal Research Institute (ARI), Mansoura branch. Fish were randomly distributed in fully prepared glass aquaria (80 × 35 × 40 cm) filled with approximately 70L of dechlorinated tap water kept at 20 °C and supplied with electrical aerators, an underwater filter, and a submersible Heater. Fish were fed twice daily at 3% of their body weight on a commercial diet (Uccma feed, Egypt; crude protein 32%) during acclimation for 2 weeks before starting the experiment.

### Experimental design

Fish were randomly assigned into three groups in triplicate (10 fish/aquarium tank, Static system). Groups were as follows; control (no zoospores exposure) and two challenged group; one group was used for sampling and the other one for mortality monitoring. Fish in both challenged groups were subjected to ami-momi treatment^27^, through shaking in a net for 1.5 min, and then placed back into the tanks. The challenged groups were exposed to 60 mL of *S. parasitica* zoospore suspension per group (one plastic bag/ aquarium containing 20 mL of zoospores suspension). At the start of the experiment, the heaters were unplugged. When the water temperature reached room temperature at 15 °C, the plastic bags containing the zoospores suspension were allowed to acclimate with each aquarium temperature and then mixed with the water gradually, as described in Zahran et al.^26^. The experiments terminated after 3 weeks. Fish were fed a commercial diet twice daily, excluding the day before sampling. Fish status was monitored daily for clinical signs of infection; gills and skin biopsies were examined from dead fish to confirm water mold infection by identifying broad, aseptate hyphae and sporangia with light microscopy.

### Sample collection

Six fish were sampled (2 fish /tank) from each group at weeks 1, 2, and 3 post-infection and anesthetized with buffer tricaine (MS-222, FINQUEL®, ARGENT). Blood samples were collected via venipuncture by using a 3-cc syringe and (23gauge) needle and emptied into a plain centrifuge tube to clot at room temperature (24–25 °C) and then kept at 4 °C for 4 h before centrifugation at 1198 × *g* for 10 min to collect serum and stored in Eppendorf tubes at − 20 °C till analysis. Serum samples were analyzed for C-reactive protein (CRP) and IgM. Gill homogenate was prepared by adding 0.5 g of gills to 4.5 mL of ice-cold phosphate buffer saline (Oxoid) at pH 7.4; and homogenized by homogenizer (Sigma, UK). The homogenate was then centrifuged at 1198 × g for 15 min at 4 °C (Centrikon H-401 centrifuge), and the resultant supernatant was aliquoted and stored at − 80 °C for later oxidative stress and antioxidant enzyme assays. Gill tissues were collected in RNAlater® solution and kept at − 80 °C until gene expression analysis. Samples of skin and gills were excised from experimentally infected fish and placed in 10% neutral buffered formalin for histopathological examination.

### Innate immune parameters

*Serum C-reactive protein* (CRP) was determined using the Rapid latex agglutination test (Biomed-Egypt), according to Sarikaputi et al.^28^. Serum IgM (Biotecnica-Italy) was estimated spectrophotometrically (BM Co. Germany, 5010) based on turbidimetric measurement according to Dati et al.^29^, following the instructions given by the supplier.

### Oxidant/antioxidant parameters

Oxidant/antioxidant enzymes were measured according to manufacturer instructions (Biodiagnostics, Egypt). Malondialdehyde (MDA) level in gill homogenate was measured spectrophotometrically (Photometer 5010, Photometer, BM Co. Germany) and represented as nmol/g tissue, according to Satoh^30^. Catalase (CAT) activity was assayed by measuring the reduction of hydrogen peroxide concentration at 240 nm, according to Aebi^31^. Reduced glutathione (GSH) level was measured using diagnostic kits, according to Beutler^32^, using Elmann's reagent (DTNB). Superoxide dismutase (SOD) activity was expressed as U/g tissue and measured by the enzymatic colorimetric method; according to Nishikimi et al.^33^, the reaction relies on the enzyme's ability to inhibit the phenazine methosulphate mediated reduction of nitroblue tetrazolium dye.

### Total RNA Extraction, cDNA synthesis, and cytokines gene expression analyses

Gene expression analyses were performed by RT-qPCR. Total RNA was extracted from gills samples (50 mg); using RNeasy Mini Kit (Qiagen) according to the manufacturer's instructions. The purity and quantity of the resulting RNA were confirmed spectrophotometrically by measuring the optical density at 260/280 nm. One µg was reverse transcribed from total RNA using iSCRIPT® (Bio-Rad) cDNA synthesis kit per user's manual; and stored at − 20 °C before qualitative and quantitative PCR analysis. All qPCR reactions were duplicated using a CFX96 Touch Real-Time PCR detection system (BIO-RAD) with SsoAdvanced Universal SYBR Green Supermix Kit to quantify gene expression levels in the tissue samples. The primer used for real-time PCR was described previously by Zahran et al.^34^. The expression of pro-inflammatory cytokines was first normalized to that of β-actin and presented as a fold change by calculating the average expression level of the infected fish gills exposed samples divided by that of the controls as described previously^35^.

### Histopathological examination

Tissue specimens of skin and gills were excised from experimentally infected fish, placed in 10% neutral buffered formalin fixative, and processed for dehydration with ascending grades of ethanol (Al-Gomhoureia Company., Egypt), cleared in xylol, and samples were then embedded in paraffin wax. Sections 5.0 microns thick were cut by microtome, mounted from a water bath into a clean glass slide, and stained with Haematoxylin and Eosin (H&E) and Periodic acid Schiff (PAS)^36^.

### Statistical analysis

All data and variances were checked for homogeneity and normality using the Levene test and Shapiro–Wilk. Data were expressed as Mean ± S.E. Statistical analysis was performed using the software SPSS (version 22, windows 7). Expression fold units were calculated by dividing the normalized expression values of the *S. parasitica* challenged group by that of the respective control group at each timepoint. Normalized individual fold change values were anchored to the lowest value recorded in each data set and then Log2 trans-formed, as described previously^37^. The significance of the average fold change between uninfected and infected groups was analyzed by one-way analysis of variance (ANOVA) followed by Duncan's multiple comparisons of group means. Differences were considered statistically significant when *p *< 0.05. Data were graphically presented using Microsoft Excel 2016.

### Ethics approval and consent to participate

All fish in the experimental protocols were reared and handled following the local Administrative Panel on Laboratory Animal Care and Committee of Mansoura University guidelines, which specifically approved this study. All experiments were performed following ARRIVE guidelines and regulations.

## Results

### Fish experimental infection with *Saprolegnia*

**Figure 1:**
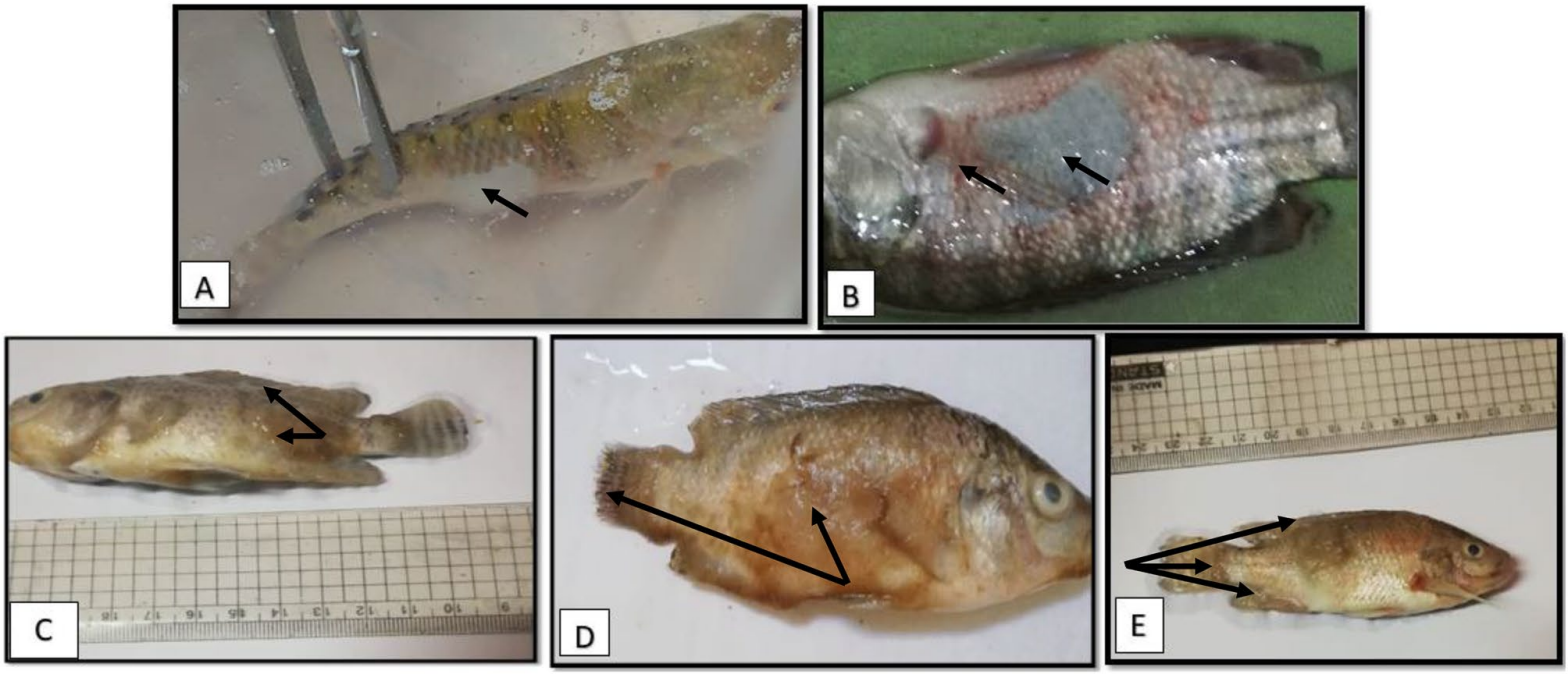
Experimentally infected Nile tilapia with *S. parasitica* showing (**A**) In aquarium: cotton wool like masses on lateral body surface and fins. (**B**) Cotton wool like masses on lateral body surface with hemorrhagic ulcer on skin; (**C**) cotton wool like masses on lateral body surface and fins; (**D**) excessive growth of cotton wool like masses on lateral body surface and loss of caudal fin; (**E**) cotton wool like masses on fins (dorsal, caudal and anal fins) and lateral body surface.

**Figure 2:**
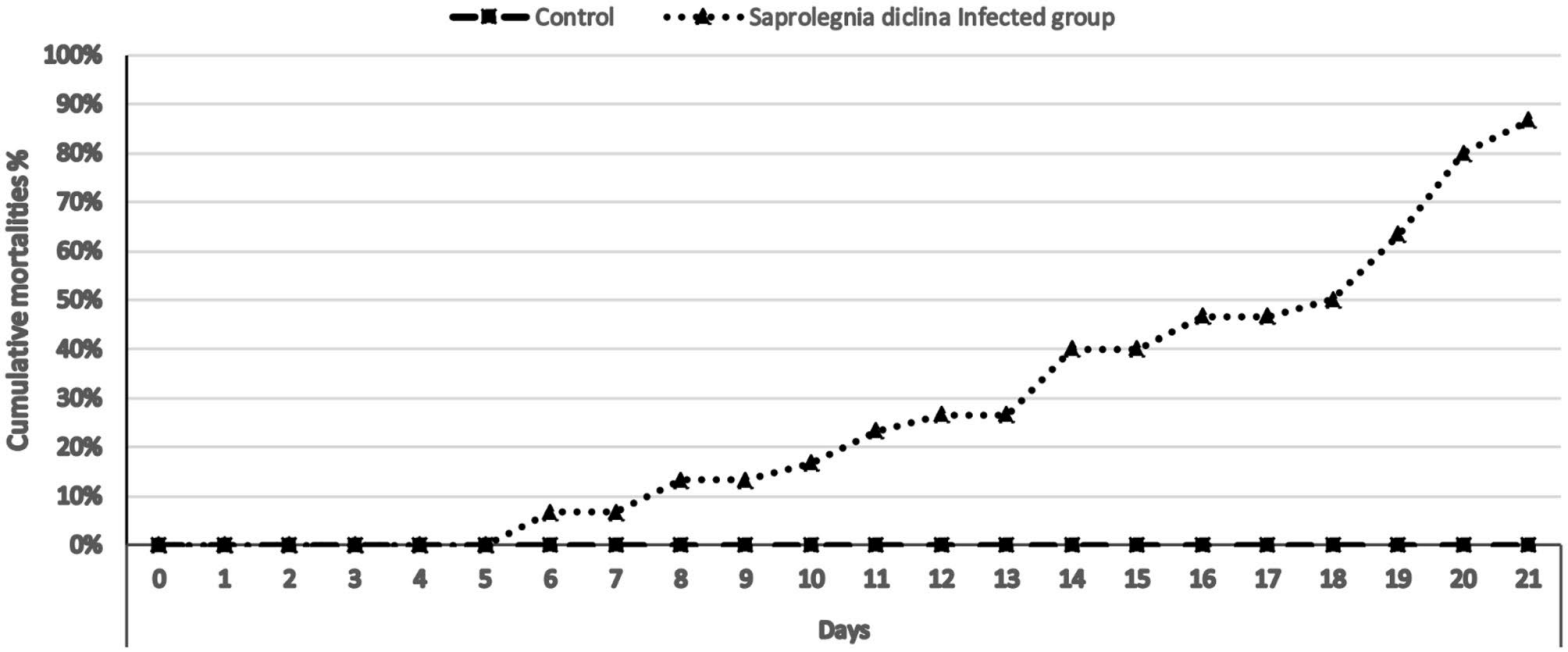
Cumulative mortality percentage among Nile tilapia post-challenge with *S. parasitica*.

The infected group with *S. parasitica* zoospores exhibited the characteristics signs of saprolegniasis, including cotton wool-like growths on the skin, fins, and lateral body surface (Fig. 1). Cumulative mortalities were higher in the infected group by up to 86.6% compared with the control non-infected one (Fig. 2).

### Innate immune parameters

**Figure 3:**
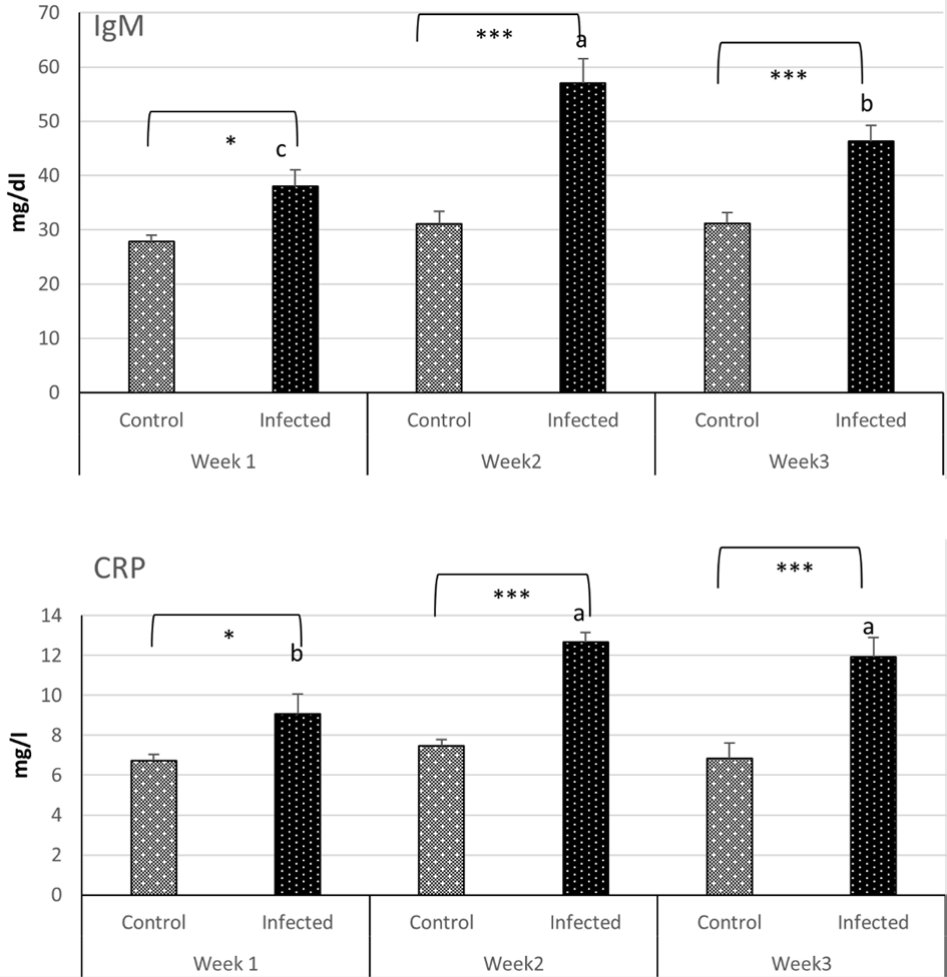
CRP and IgM response in serum of Nile tilapia fish challenged with *S. parasitica* zoospores compared to control group. (n = 6 fish/group). Values are reported as mean ± SE. Values with a different letter and asterisk superscript are significantly different between and within groups respectively (*p* < 0.05).

IgM and CRP levels showed the same trend, since they exhibited a significant increase in the infected group at week 1 (*P* < 0.05), 2, and 3 (*P* < 0.0001) compared to the control group. Interestingly, both levels increased significantly over weeks 2 and 3 compared to week 1 in the infected group (Fig. 3).

### Oxidant and antioxidant parameters (MDA, CAT, SOD, GSH)

**Figure 4:**
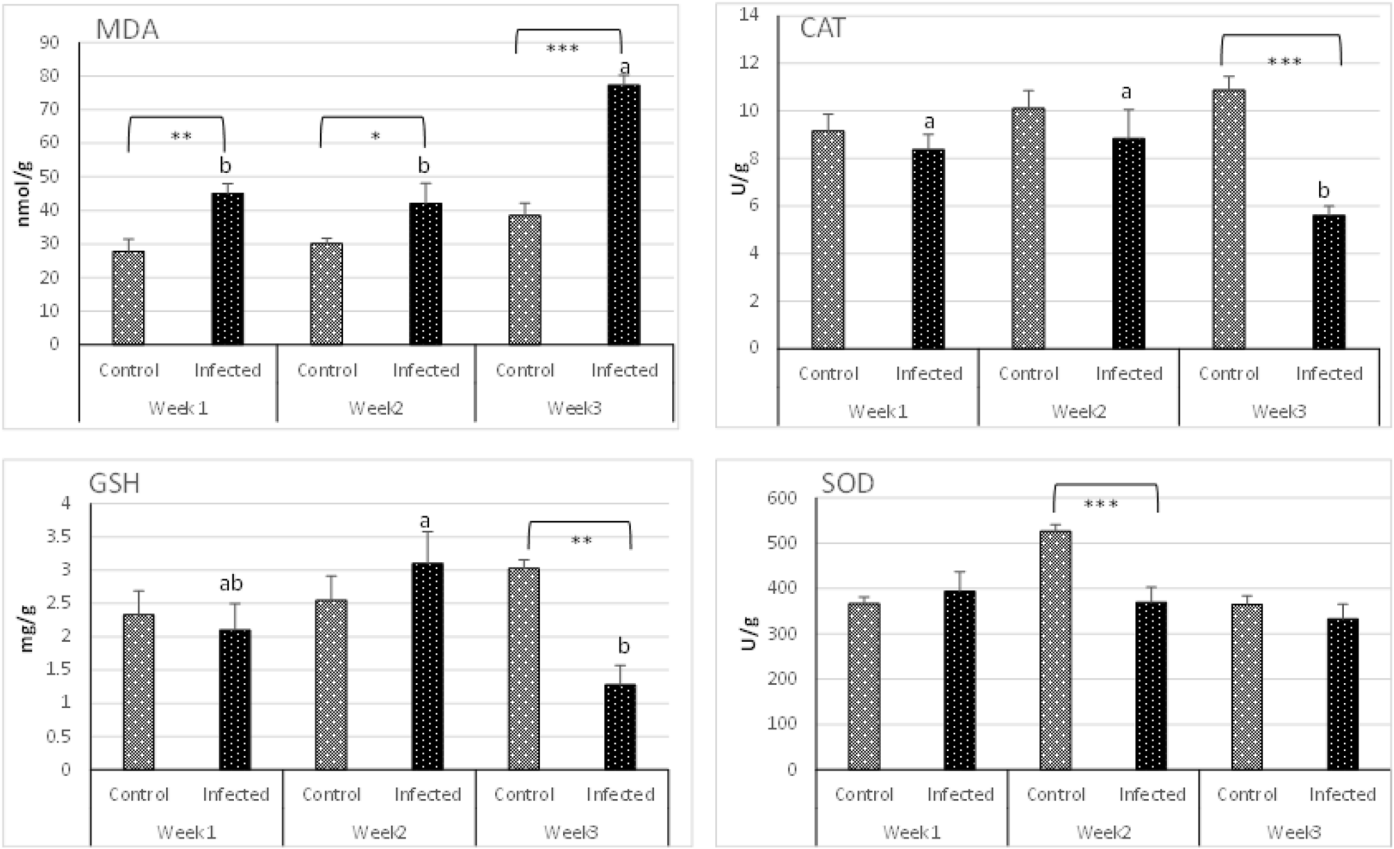
Branchial MDA, CAT, GSH, SOD levels in Nile tilapia challenged with *S. parasitica* zoospores compared to control group. N = 6 fish/sampling time. Values are reported as mean ± SE. Values with a different letter and asterisk superscript are significantly different between and within groups respectively (*p* < 0.05).

MDA level was significantly elevated in the infected group at week 1 (*P* < 0.001), 2 (*P* < 0.05), and 3 (*P* < 0.0001) compared to the control group. MDA level increased significantly over time, showing a significant increase at week 3 (*P* < 0.0001) compared to weeks 1 and 2 in the infected group, with no statistical changes between the latter. CAT level significantly decreased at week 3 (*P* < 0.0001) in the infected group compared to the control group. Additionally, CAT level was reduced considerably in the infected group at week 3 (*P* < 0.001) compared to weeks 1 and 2 with no statistical changes between the latter. GSH level was in line with CAT level, significantly decreasing at week 3 (*P* < 0.001) in the infected group compared to the control. Meanwhile, the GSH level exhibited a significant decrease at week 3 (*P* < 0.001) compared to week 2. SOD level was significantly decreased in the infected group at week 2 (*P* < 0.0001) compared to the control. However, no other statistical changes in SOD level were observed neither between the infected and control group nor among the infected group over time (Fig. 4).

### Immune gene expression

**Figure 5:**
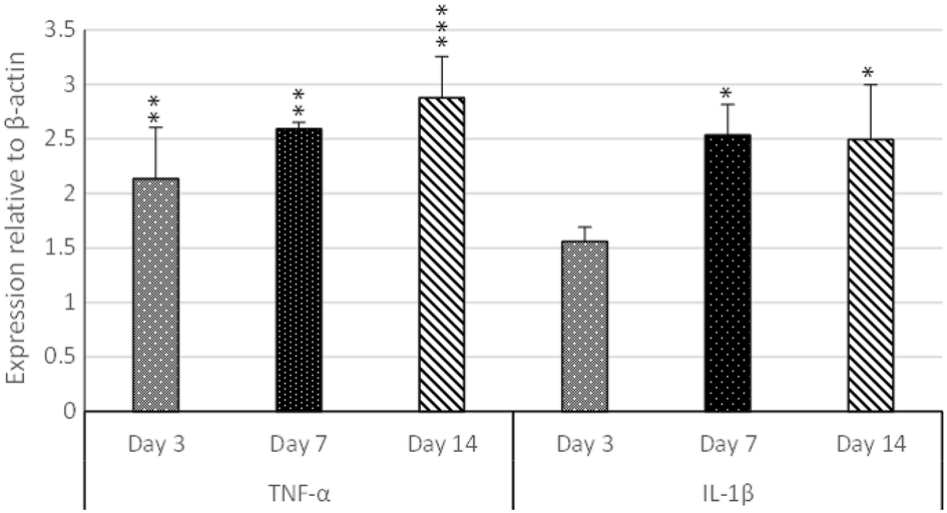
Branchial TNF-α and IL-1 β mRNA levels in Nile tilapia challenged with *S. parasitica.* mRNA expression patterns of selected genes (TNF α and IL-1 β) were analyzed at 3, 7and 14-day post infection (dpi). Data are presented relative to that of un-challenged fish (control). Significant differences were defined at *p* < 0.05 and all data are represented as mean ± SE.

TNF-α gene was significantly upregulated in branchial tissue at all-time points, particularly at day 14 (~ threefold, *P* < 0.0001) post-infection compared to the control. Meanwhile, the IL-1 β gene was significantly upregulated (*P* < 0.05) only at days 7 and 14 (2.5 fold) post-infection compared to the control (Fig. 5).

### Histopathological examination

**Figure 6:**
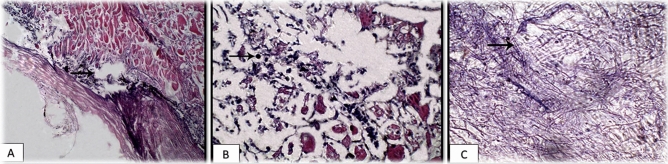
Skin section of experimentally infected Nile tilapia with *S. parasitica* showing (**A**) marked edema, extended to the underlying musculature (arrow) (H&E, × 100); (**B**) marked edema, round cells recruitment (arrow) and myocytolysis in the underlying musculature (H&E, × 400); (**C**) Reddish-stained hyphae (arrow) of *Saprolegnia* (PAS, × 400).

**Figure 7:**
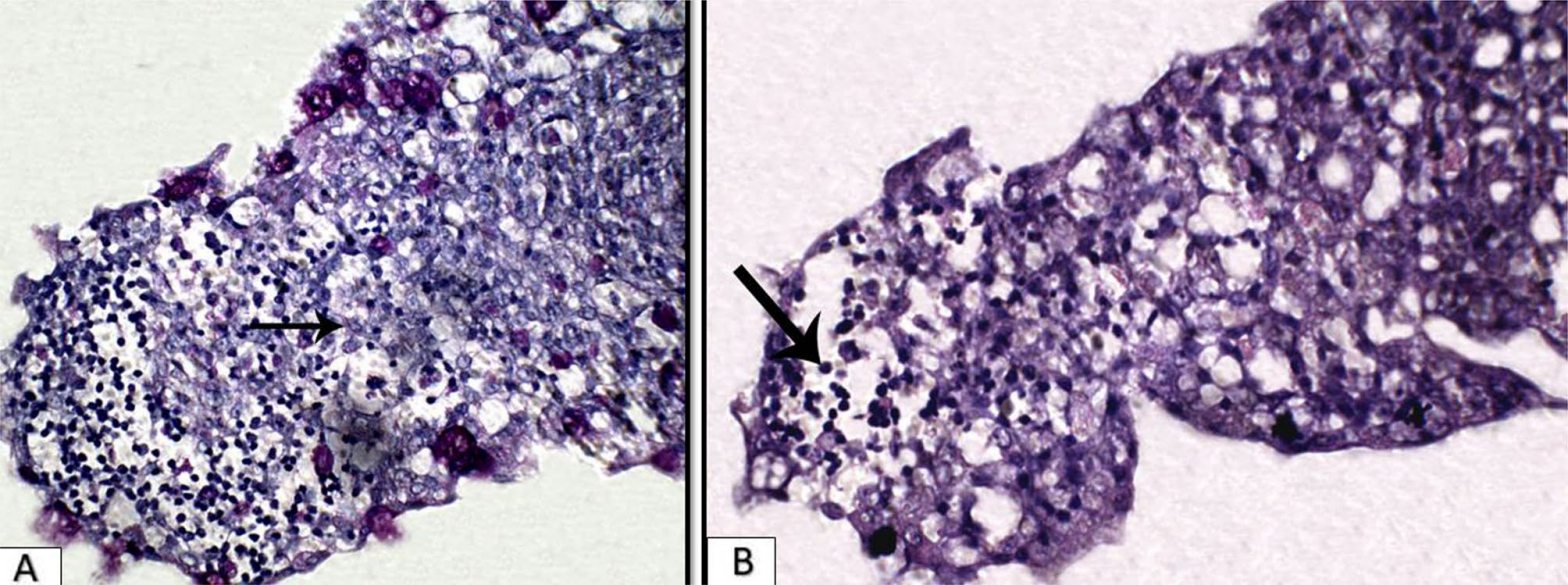
Gill section of experimentally infected Nile tilapia with *S. parasitica* showing, (**A**) marked edema and round cell recruitment into primary lamellae (arrow) with goblet cell metaplasia (H&E, × 400); (**B**) Gill section showing reddish stained hyphae (arrow) suspected to be fungal hyphae of *Saprolegnia* (PAS, × 400).

Histopathological findings of the skin revealed desquamation of the superficial layer of skin with heterophilic and round cell infiltration and marked edema that extended to the muscle beneath. In addition, round cell recruitment and myocytolysis in the muscle underneath were noticed (Fig. 6A–C). The gills exhibited marked edema, round cell recruitment into primary lamellae, and goblet cell metaplasia (Fig. 7A,B).

## Discussion

In the current study, *S. parasitica* was pathogenic to Nile tilapia, leading to an 86.6% mortality rate. The clinical signs were similar to those reported in previous studies^38–42^. Elevated fish mortality might be attributed to the failure in the osmotic balance (hemodilution) and respiratory failure following massive destruction of the epidermis and degeneration of secondary lamella by invasive hyphal growth, histopathologically evidenced. Fish oomycetes with different virulence factors that attack host tissue as extracellular effectors (proteases, gluconases, and hydrolases) are secreted by pathogens into the host extracellular space, altering the host-cell structure and function. Extracellular effectors can be subdivided into two major categories: effectors mediating protection against host defense and effectors mediating invasion. These hypotheses have been supported by other studies, which discovered that oomycetes secrete hydrolytic proteins (glycosyl hydrolases) into the extracellular space, which aid in the breakdown of cell wall components and thus allow entry into host tissues^43,44^. Additionally, intracellular effectors proteins (RxLR-effectors, host-targeting protein 1 (SpHtp1)) translocated by Oomycetes into the host cell, where it modulates molecular processes in their hosts to suppress immune responses and thereby help to establish an infection^45^ with subsequent mortalities.

Similar to our findings, Zaki et al.^46^ noticed Nile tilapia experimentally infected with *S. parasitica* zoospore (4 × 10^6^ zoospores/L) exhibited typical water mold infection symptoms seven days after infection. In addition, Nile tilapia exposed to *S. ferax* zoospores (2 × 10^4^ spore/L) showed cumulative mortalities of up to 92.5% over 3 weeks^47^. Stueland et al.^38^ revealed 89 and 31% cumulative mortalities when Atlantic salmons, *Salmo salar L. S. declina* zoospore (2.5 × 10^6^ spore/mL) experimental infection in scaled carp fingerling revealed 55% cumulative mortalities^48^, *S. parasitica* spores at a concentration of 1.0 × 10^4^ zoospores/L were able to induce 80% mortality ten days following challenges^42^, and Kumar et al.^49^ recorded 100% mortality in *Pangasianodon hypophthalmus* experimentally infected with *S. parasitica* zoospores (2 × 10^6^/L).

The CRP is one of the several proteins often referred to as acute phase reactants that play vital roles in various defense-related activities, particularly with the immune and chemical detoxifying systems, including inflammatory response, prevention of the infection spread, and restoration of the healthy state^50,51^. In the current study, CRP level was significantly increased in the infected group at all-time points compared to the control and elevated (*P *< 0.05) in the infected group over time, which is confirmed histopathologically. Our results confirm the role of CRP in mediating the complex inflammatory response, limiting the dispersal of infectious agents, inactivating proteases, killing microbes, repair of tissue damage, and restoring the healthy one. All this suggest CRP as a bioindicator in the pathogenesis of fish infectious diseases^52^. Similar to our findings, the level of serum CRPs increased significantly in common carp (*Cyprinus carpio*) following infection with *A. hydrophila*^53^ and in Nile tilapia experimentally infected with *Streptococcus inia* at 7, 14, and 21-day post-infection^52^ and in Pacu, *Piaractus mesopotamicus* experimentally challenged with *A. hydrophila* (1 × 10^8^) at day seven post infection^54^.

IgM is an innate immune defense in fish. It acts synergistically with other soluble molecules, including anti-proteases, lysozyme, complement, transferrin, interferon, and C-reactive protein, to protect fish against infections^16–18,55^. However, the adaptive response initiated through a complex network of cells, genes, proteins, and cytokines stimulates the host response to the antibodies and antigens^18,56^. IgM level in the current study exhibited a similar trend as CRP, which might be attributed to the contribution of IgM in the defense mechanism against microbial infection^57,58^, through neutralizing specific antigens, activating the complement system, agglutination, binding of mannose-binding lectin and mediating cellular cytotoxicity^57–59^. Our results coincided with Li et al.^17^, who found that the highest level of IgM was recorded at day seven post-infection with *A. hydrophila* in grass carp. Additionally, Yin et al.^60^ found that IgM concentration was significantly increased in serum and tissue supernatant after *Streptococcusagalactiae* (ZQ1901) infection in tilapia. Further, IgM gene expression increased significantly from day 6 to 14 in the head kidney, spleen, thymus gland, and blood cells of the orange-spotted grouper after infection of *Vibrio alginolyticus*^61^.

In general, oxidative stress is the consequence of either excessive production of free radicals (FRs) or a shortage in antioxidant enzymes such as SOD, CAT, and GSH. The presence of toxic chemicals, pollutants, or infections in fish environments could promote FRs production^62–66^. When the antioxidant system cannot cope with the oxidation rate of cell components, an irreversible process known as oxidative damage occurs, that is biomolecular damage caused by the attack of free radicals as reactive oxygen species (ROS)^67,68^.

Herein, the infected group's MDA level was significantly increased at all time points compared to the control. Besides, it was highly significant at week 3 compared to other time points in the infected group, which indicated an excessive production of free radicals and lipid peroxidation, both of which may have contributed to the necrosis and loss of epidermal and branchial epithelium that was observed in the histopathological examinations. This result could be explained by increased FRs production due to *Saprolegnia* infection. These FRs devastate polyunsaturated fatty acids of cell membrane lipids and cause lipid peroxidation^69^ represented in MDA levels as a biomarker of oxidative stress and cellular damage^70–72^. Our findings aligned with Azimzadeh and Amniattalab^73^, who recorded higher levels of MDA in naturally infected rainbow trout with *Saprolegnia* compared to the non-infected (healthy) group. In the same context, brown trout naturally infected with ulcerative dermal mycosis revealed a significant increase in MDA level in the spawn homogenate compared to healthy trout^74^. Similarly, Nile tilapia, infected intraperitoneally with *Enterococcus faecalis* (3 × 10^8^ CFU/mL), demonstrated a significant increase in serum MDA level at days 7 and 14 post-infection^34^. Further, Nadirah et al.^75^ found that plasma MDA level was significantly increased in red hybrid tilapia experimentally infected with *S. agalactiae* (2.3 × 109 CFU/mL) under influences of heat stress at 33 ± 0.5 °C.

CAT and GSH levels were significantly decreased in the infected group compared to the control at week 3 and to other infected groups at the other time points. Additionally, the SOD level was significantly decreased in the infected group compared to the control group at week 2. The increased utilization of these antioxidants could explain our finding to counteract the degenerative effect of saprolegniasis. SOD acts on the superoxide anion transforming in H_2_O_2_, avoiding the accumulation of O2-, which is highly reactive and degenerative to the cell; the produced H_2_O_2_ act as a substrate for CAT enzyme to be converted into H_2_O^17,54,76,77^. Our results are consistent with those of Ali et al.^76^, who recorded that Nile tilapia fish infected by *A. laevis* and *P. herbarum* showed a pronounced drop in CAT activity in the muscle and gill tissues. However, the SOD level showed a significant elevation. In another study, CAT was considerably reduced with no SOD changes in naturally infected rainbow trout by *Saprolegnia* compared to healthy ones^73^. In Zebra fish experimentally infected with *S. parasitica* both SOD and CAT levels were lowered^78^. In the same context, a significant reduction of CAT, levels was reported in two species of shrimps after pathogen infections: *Penaeus monodon* infected with white spot syndrome virus (WSSV)^79^ and *Litopenaeus stylirostris* infected with *Vibrio nigripulchritudo*. Baldissera et al.^68^ and Zahran and Risha^47^ recorded a significant inhibition of liver and serum SOD activity in grass carp and Nile tilapia naturally infected with *S. parasitica and S. ferax* found; respectively indicating lower protection against oxidative damage induced by superoxides^70^. Several studies reported drop in GSH activity in muscles gills, serum, head, kidney and liver of Nile tilapia naturally or experimentally infected with various pathogens such as *A. laevis* and *P. herbarum*^76^, *E. faecali*^34^ and *Falvobacterium colmnare*^80^*.* However, Zahran and Risha^47^ found that GSH activities were significantly higher after the challenge of Nile tilapia with *S. ferax* zoospores. This result could be attributed to higher excretion of GSH in response to the produced ROS in the early stage as a compensatory mechanism due to the damage induced by infection.

The expression of cytokine genes is an effective tool for evaluating the immune response^21^. These cytokines trigger the immune system and induce inflammatory responses; they are also involved in different functions of the fish immune system, such as regulation, increasing the growth and function of immune cells^22,81^. Pro-inflammatory cytokines play a crucial role in the defense against microbial pathogens, including *Saprolegnia*. IL-1β, in particular, seems to play an essential role in *S. parasitica* infection; it stimulates the innate immune system and affects T and B cell activation^82,83^. *TNF-α* is a critical component in early inflammatory events synthesized by either different types of cells through stimulation by endotoxins, inflammatory mediators, and cytokines such as *IL-1* or following stimulation by TNF itself^84,85^.

In our study, *TNF- α* gene was significantly upregulated in gills at all-time points, particularly at day 14 post-infection, compared to the control. Meanwhile, the *IL-1β* gene was upregulated significantly only at days 7 and 14 post-infection. This finding could be explained due to the roles of both *TNF- α,* and *IL-1β* in the host responses to bacterial attack, tissue damage, and immune response involved in autoimmune diseases^84,86^. The induction and activation of various oxidant-generating enzymes in inflammatory cells are regulated by pro-inflammatory cytokines, such as *TNF- α*, *IL-1*, *IL-4*, *IL-6,* and interferon-gamma (*INF-γ*). Thus, CRP and the oxidative stress results confirmed cytokine production, which subsequently activates enzymes producing different free radicals and oxidants^20^. Our results coincided with previous ones obtained in different species of fish^82,83^, where a strong up-regulation of *IL-1β* was evident in RTS11, RTL, and RTS11 cells in response to *Saprolegnia* infection. Moreover, Shin et al.^78^ recorded an upregulated expression pattern of pro-inflammatory cytokine and chemokines (*IL-1β* and *TNF-α*) at three and 12-h post-infection in Zebra fish experimentally infected with *S. parasitica*. Also, Beckmann et al.^87^ found that a significant stimulation of (*IL-1β* and *TNFα*1) genes was demonstrated within 6 h for vaccinated Atlantic salmon. Stimulating inflammatory gene expression is a typical response in fish to vaccination with bacterial antigens and infection with bacteria, viruses, and parasites^87,88^. Moreover, Belmonte et al.^23^ concluded that *Saprolegnia* triggers a robust inflammatory response in Atlantic salmon (i.e., induction of *IL-1β*, *IL-6*, and *TNF- α*) and in Nile tilapia experimentally infected with *S. agalactiae* (Group B *streptococci*; GBS) between 6 and 96 h post-infection^89^.

In general, histopathological changes can be caused by the indirect or direct activity of pathogens on tissues. Molecular, biochemical, and physiological changes are associated with indirect action^90^. As previously mentioned, the histopathological lesions in the studied organs indicate other findings, such as the release of reactive oxygen species (ROS) or the diminished antioxidant defense and the elevation of pro-inflammatory cytokines. Similar to previously published results on *Saprolegnia* experimentally infecting fishes, the present investigation revealed extensive loss of the epidermis, degenerative alterations in the dermis and muscle fibers, and secondary lamellar degeneration in infected fish^49,91,92^.

## Conclusion

To conclude, we have investigated particular arrays of biomarkers following infection with *S. parasitica*. Our findings suggest that the upregulation of such biomarkers potentially plays a vital role in the defense of Nile tilapia against *S. parasitica.* The present work therefore provides insights into the immune responses of Nile tilapia infected with *S. parasitica*, that are mostly in line with those already published in other fish species infected by various pathogens. Our study is therefore helpful in the identification of possible biomarkers associated with disease status in Nile tilapia, possibly in early stages of the infection. The lack of treatments to protect fish from saprolegniasis in aquaculture, however, and the high mortality observed after these infections makes it urgent to clarify additional anti-*Saprolegnia* defenses in fish.

## Data Availability

The data supporting this study's findings are available from the corresponding author upon reasonable request.
